# Effects of atmospheric pressure plasma jet operating with DBD on *Lavatera thuringiaca* L. seeds’ germination

**DOI:** 10.1371/journal.pone.0194349

**Published:** 2018-04-09

**Authors:** Joanna Pawłat, Agnieszka Starek, Agnieszka Sujak, Piotr Terebun, Michał Kwiatkowski, Małgorzata Budzeń, Dariusz Andrejko

**Affiliations:** 1 Institiute of Electrical Engineering and Electrotechnologies, Lublin University of Technology, Lublin, Poland; 2 Department of Biological Bases of Food and Feed Technologies, University of Life Sciences in Lublin, Lublin, Poland; 3 Department of Biophysics, University of Life Sciences in Lublin, Lublin, Poland; Universite Toulouse III Paul Sabatier, FRANCE

## Abstract

The paper presents the results of an experiment on the effect of pre-sowing stimulation of seeds with atmospheric pressure plasma jet operating with dielectric barrier discharge (DBD plasma jet) on the process of germination of Thuringian Mallow (*Lavatera thuringiaca* L.). Five groups of seeds characterized by a different exposure times (1, 2, 5, 10 and 15 minutes) as well as untreated seeds—control were used. Pre-sowing plasma stimulation of seeds improved germination parameters such as: germination capacity and germination energy for all tested groups relative to control. The highest germination parameters were obtained for seeds stimulated with plasma for the exposure times of 2 and 5 min. The analysis of the contact surface angle indicated the decrease of its’ mean values upon seed stimulation while no statistical effects were observed. Analysis of the SEM scans revealed the increase in seed pattern intensity which could be attributed to removing of the surface parts of cuticle possibly covered with wax upon short time—2 and 5 min plasma treatment. Such a phenomenon can act similarly to mechanical scarification of seeds. Longer exposure of seeds to plasma resulted in affecting the deeper zone of cuticle and damage or fracture of some parts of the cuticle. Lower germination parameters of seeds upon longer exposure times to plasma may indicate mechanical damage of the seeds.

## Introduction

Thuringian Mallow (*Lavatera thuringiaca* L.) from the family Malvaceae is a self-pollinated, perennial plant with numerous stiff stems attaining the maximal height of 150–220 cm. In a maturity stage seed-vessel containing about 20 seeds is formed. The Uleko cultivar examined in this study is a melliferous plant that produces up to 8.8 mg nectar per day per flower (100–150 kg/ha of honey) [1, 2]. It can be used for feed [3] and is suitable for the production of biomass energy. It can be applied as a source of raw material for the pulp and paper industry [4] and in the pharmaceutical industry (contains rhamnose and arabinose) due to its anti-inflammatory properties. In a long-term perspective this is agriculturally important plant because of the combination of features like frost-resistance and low demand for water, which makes it suitable for cultivation on set-aside soils. *Lavatera* is not very popular because seeds of this plant are characterized by low germination capacity under natural growing conditions. However, it is an important species for enhancement of plant biodiversity in Europe. That is why environmentally safe methods to improve the germination parameters of this plant are investigated.

Very few publications on pre-sowing stimulation of *Lavatera* seeds exist. Kornarzyński et al. (2015) has stated the positive effects of alternating electric field and magnetically treated water on germination of *Lavatera thuringiaca* L. seeds [5]. In the same study, the authors have observed that alternating magnetic field negatively affected the germination of seeds of *Lavatera*. Authors have demonstrated that the influence of physical factors on seed germination can vary and be determined by factors such as exposure dose, species and variety, or seed humidity. On the other hand, field experiments on the pre-sowing laser stimulation conducted on *Lavatera* seeds have shown the increase of its field emergence which could possibly facilitate the acceleration of the germination process [5].

Non-thermal plasma has been used for biological and chemical decontamination including variety of applications such as gas and water purification, biological and chemical decontamination of surfaces including removal of bacteria and biofilms from inanimate surfaces and also from biological materials such as skin and wounds [6–33]. Low temperature plasma has been applied for modification of material surfaces to change water surface angle, to improve coating properties, to preserve archeological objects, etc. [34–46]. Non-equilibrium plasma has been used to decontaminate seeds from pathogens, to improve their germination properties and length of sprouts [47–61]. Enhancement of spinach seed germination and seedling growth after micro DBD plasma treatment has been observed by Ji et al., 2016 [62]. Air plasma has shown to act slightly better than pure N_2_ plasma. Kitazaki et al. [47] from group of Shiratani and Hayashi has applied low pressure and atmospheric pressure plasmas to successfully enhance length of sprouts of radish, rice, plumeria and zinnia. Authors have pointed the main role of radicals correlated with O_3_ and NOx (nitrogen oxides) concentration, strongly indicating that optimum conditions depended on the species. They have also claimed the negligible influence of ions and photons [53, 60]. Ono et al., 2017 have investigated positive inactivation effect of low-pressure RF oxygen and air plasmas on cabbage seed-borne bacteria: *Xanthomonas campestris pv*. *campestris* (Xcc) on plant seed surfaces [49]. Puligundla et al., 2017 have shown 1.2–2.2 log CFU/g reduction of *Bacillus cereus*, *Escherichia coli*, *Salmonella spp* on rapeseed seeds after 3 min treatment with corona discharge plasma jet and positive influence of treatment times up to 2 min on germination rate and seedling growth [61]. Dobrin et al., 2015 has applied surface discharge reactor at atmospheric pressure to treat wheat seeds. According to the authors this treatment have influenced the growth parameters resulting in longer and heavier sprouts as compared to control sample but plasma in this case had little effect on the germination rate [58]. Plasma based treatment methods have been considered environmentally safe as used reactors were low energy consuming, the treatment process did not cause additional contamination, amounts of generated oxidants were low and they did not retain in the ecosystem [63].

The aim of this study was to evaluate the impact of DBD plasma jet applied prior to sowing on the process of germination of Thuringian Mallow seeds (variety Uleko).

## Materials and methods

Experimental material consisted of *Lavatera thuringiaca* L. seeds variety Uleko from Plant Breeding and Acclimatization Institute, National Research Institute (Radzików, Poland) collected in 2009 year.

**Fig 1:**
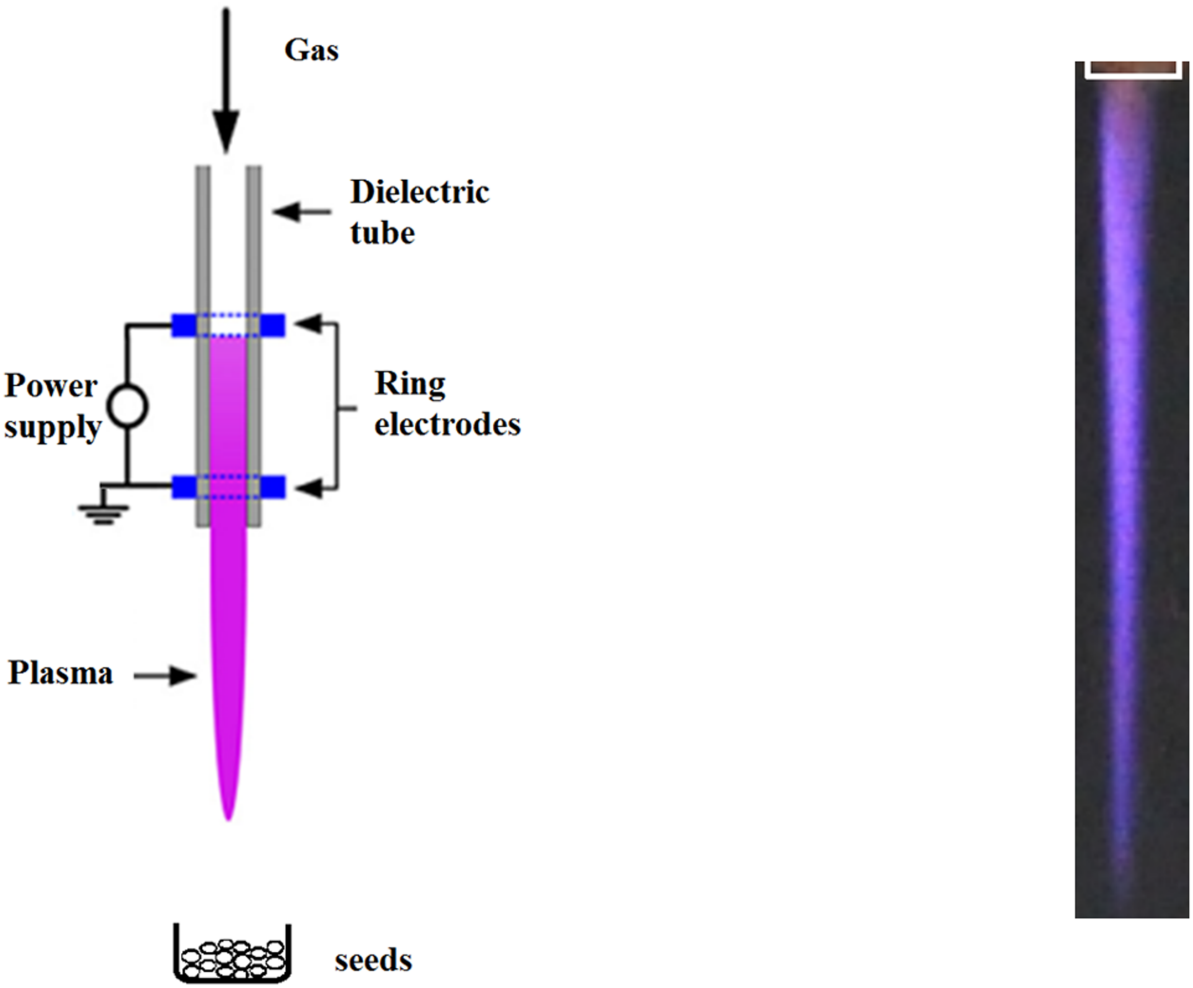
Experimental set-up with mini DBD plasma jet reactor (A) and the photo of the discharge (B).

Plasma was generated in set-up with dielectric barrier discharge (DBD) plasma jet, as depicted in Fig 1. The ceramic tube of jet had internal and external diameters of 1.4 mm and 3.4 mm, respectively. Distance between two copper ring electrodes was 12 mm. The flow of the gas mixture of 1.6 dm^3^/min of helium with 0.03 dm^3^/min of nitrogen was adjusted by gas flow controllers.

Reactor was supplied by voltage of 3.7 kV with frequency of 17 kHz and mean power of 6 W. Temperature was measured using uninsulated K-type thermocouple with electronic temperature compensation multimeter. Open container with the seeds was placed in ambient air under the outlet of plasma jet. Distance between plasma jet and sample was 5 cm and the highest temperature registered on the seeds surface was 40°C. Mechanical mixing of seeds was not applied, however as the size of seeds was relatively small, they were naturally mixed by the outlet gas flow from the plasma jet. Concentration of ozone was measured above the seeds with use of Ozone ECO-Sensor A-21ZX.

Experiment on seed germination was carried out under laboratory conditions, i.e. lighting E = 360±5 lux (period 12/12) and temperature T = 25°C±2°C. Samples of 100 pieces of seeds were placed on Petri dishes lined with four layers of filter (blotting) paper. Experiments were performed in four replicates.

Five groups of seeds characterized by a different exposure times to plasma were used (1, 2, 5, 10 and 15 minutes) as well as a control—untreated seeds. All groups of seeds were watered with the same amounts of double-distilled water.

Number of sprouts was determined every 24 hours. Fraction of germinated seeds (number of sprouts) after 10 days of germination was defined as germination energy *G*_*EN*_, while fraction of germinated seeds after 21 days of germination was defined as germination capacity—*G*_*C*_ (ISTA 2012) [64]. Both germination energy and germination capacity were expressed as a fraction of the germinated seeds *G* after a certain time *t* and calculated from the following equation:

$$G=\frac{n}{n_{T}}\cdot 100\%$$ (1)

where: *n*—the number of seeds germinated at time *t*, *n*_*T*_—the total number of sown seeds.

The surface contact angle was measured using Kruss DSA25E goniometer equipped with CCD camera. Contact angle was studied through the sessile drop method (0.5 μl of pure water droplet) using static contact angle measurements. The experiments were performed at room temperature (25°C) by placing a liquid drop onto the surface of the seeds. The images selected for calculations were recorded in the normal mode after drop stabilization. The Young/Laplace equation (implemented in the instrument software) was used to fit each image, in order to obtain a contact angle value. The value of the contact angle, characteristic to the surface, was obtained by averaging the mean contact angles (in five independent measurements) and the error was estimated by the standard deviation of these values.

Effect of DBD plasma jet treatment was examined by the analysis of the microscopic pictures of carbon coated seed’s surfaces performed with application of the electron scanning microscope QUANTA FEG 250 with energy dispersed spectroscopy analysis. Measurements were performed at high vacuum and at various magnifications (100x, 500x, 1000x, 4000x). The accelerating voltage for the electrons (HV) was 10 kV, working distance (WD) ranged from 10.6 to 11 mm, the horizontal field width (HFW) ranged from 74.6 μm (for magnification of 4000x) to 2.98 mm (for magnification of 100x).

StatSoft—Statistica 8.0 was used for the analysis of the obtained data. Statistical differences between groups were examined with use of one-way analysis of variance (ANOVA). Tukey’s test was used to analyze the significance of differences between mean values (α ≤ 0.05).

## Results and discussion

Plasma can act on the biological material via impingement of charged particles. During operation of reactor, while plasma generation the ions and electrons, free radicals, ozone, nitrogen oxides and hydroxyperoxide are formed. Physical factors such as electromagnetic field, visible, ultraviolet and infrared radiation and also effects of gas flow such as shear stress and drying are also noted [65]. Non-thermal plasma generated at atmospheric pressure in the DBD plasma jet affected seeds’ samples indirectly. Electrical current flew only between the electrodes of the reactors but sample was influenced rather by longer living, neutral species directly generated in plasma or active species evolved from radicals and charged species, which were primarily generated in the plasma. Nitrogen plasma has radiation emissions in UV region, which can influence the biological material [66, 67] however in this case admixture of nitrogen gas was relatively low. On the other hand active species formed in the discharge zone could react with ambient air forming reactive oxygen and nitrogen species. In the outlet of the plasma reactors mostly ozone was detected (Table 1). Influence of heat was eliminated by adjusting the distance between a reactor and sample.

**Table 1:** Results of the ozone concentration measurements.

**DBD plasma jet treatment time, [min]**	00	11	22	35	110	115
**O**_**3**_ **concentration, [ppm]**	0.01	0.01	0.01	0.02	0.03	0.03

**Fig 2:**
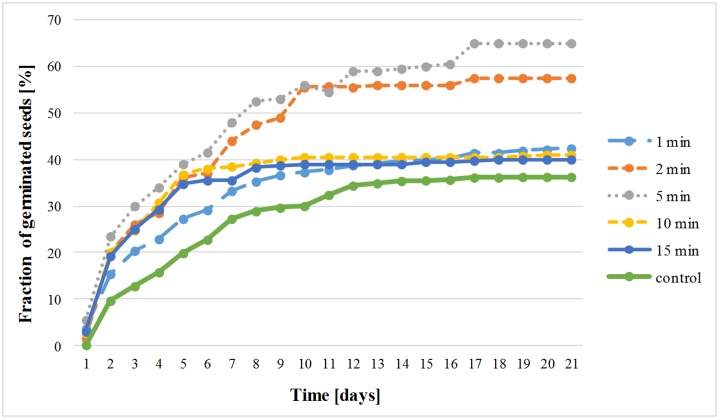
Fraction of germinated seeds of *Lavatera thuringiaca* L. after pre-sowing treatment with DBD plasma jet.

Fig 2 presents the dynamics of the process of germination of Thuringian Mallow seeds treated with plasma. Each point represents the mean value of the fraction of germinated seeds calculated on the basis of 4 independent experiments. Standard errors are not shown for the clarity of presentation. The calculated errors were within the similar range considering the certain day of experiment and amounted between 0.33 for the first day (control sample) to 14.7 for the last day (sample with the exposure time of 2 min.). The clear effect of an increase of the mean value of the fraction of germinated seeds is visible upon plasma stimulation in the case of all the exposure times.

Table 2 shows the obtained parameters of germination of the examined seeds. In the case of pre-stimulation of seeds with cold plasma generated in DBD plasma jet, the highest germination capacity of the seeds was registered for seeds after plasma stimulation for 5 min.—65%, followed by an exposure time of 2 min—61.5%. In the case of control sample this parameter amounted to 36.25%. Similarly, the highest germination energy was observed for samples with exposure times of 2 (56%) and 5 min (55.5%). This parameter was significantly higher than in the case of control seeds. On the basis of the data obtained on using DBD plasma jet for stimulation of *Lavatera* seed it is seen that the most positive effects were noted for exposure times of 2 and 5 min.

**Table 2:** The results on germination parameters of Thuringian Mallow seeds after pre-sowing treatment with cold plasma at different exposure times.

Time of stimulation, [min]	Germination energy, *G*_*EN*_ [%]	Germination capacity, *G*_*C*_ [%]
1	37.25±4.91^a^	42.38±5.23^a^
2	55.5±5.00^b^	61.5±8.70^b^
5	56±5.89^b^	65±8.72^b^
10	40.5±2.89^a^	41±3.74^a^
15	39±4.97^a^	40±5.48^a^
Control	30±2.00^a^	36.25±2.22^a^

± standard deviation

Mean values in columns marked with the same letter do not differ significantly at the significance level of α ≤ 0.05.

Based on the Tukey test of the significance of differences, time was found a factor influencing statistically germination of the Thuringian Mallow seeds stimulated with DBD plasma jet. These results indicate that seed treatment by low temperature atmospheric pressure plasma is suitable for increasing of the germination rate of Thuringian Mallow seeds.

**Fig 3:**
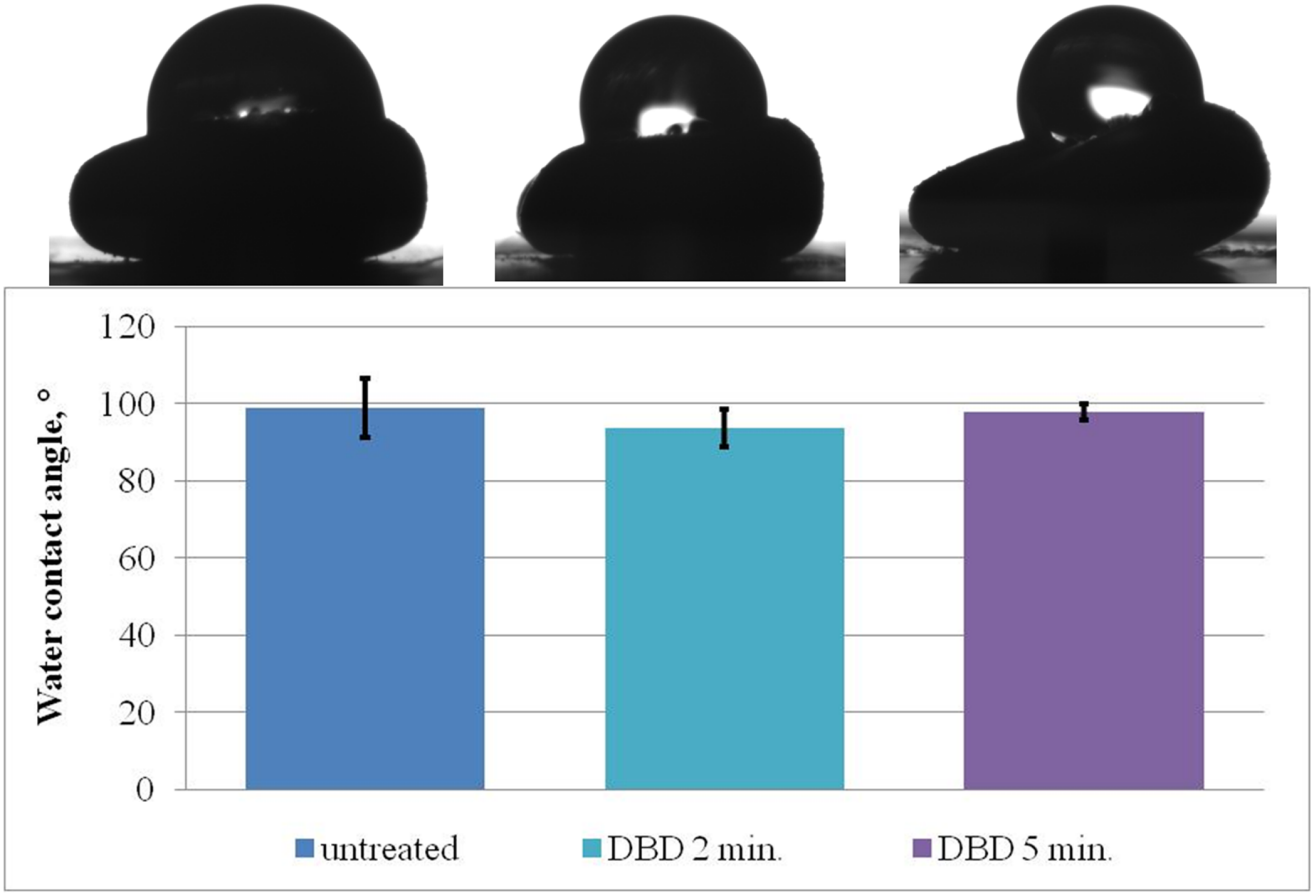
The dependence between contact angle and DBD plasma jet exposure time.

The results on the effect of DBD plasma jet treatment on water wettability are depicted in Fig 3. As Thuringian Mallow seeds are small in size and naturally highly non-uniform in shape and surface structure, the flat area of the seed was smaller or comparable to the size of the water droplet. Water contact angle values are more dependent on the wettability properties of the seeds surface than on seed’s size and shape. Although the differences in the water contact angle were not observed due to technical limitations a tendency of a decrease of the mean value of this parameter was observed.

**Fig 4:**
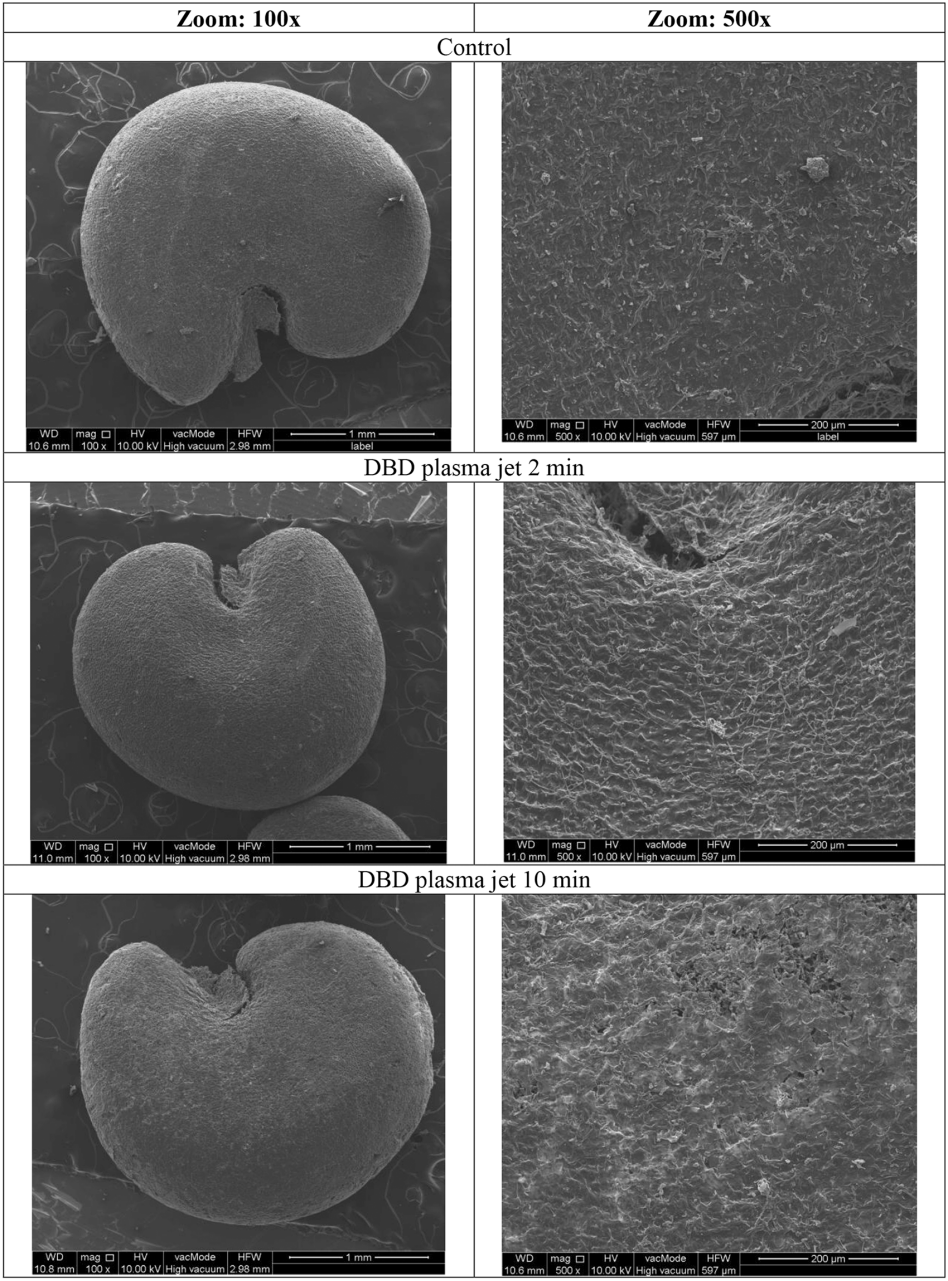
SEM photos of DBD plasma jet treated seeds of *Lavatera thuringiaca* L.

Fig 4 presents the *Lavatera thuringiaca* L seed surface scans investigated under SEM microscope at the magnification of 100x and 500x. Kidney-shaped *Lavatera* seeds show a distinctive hilum, which is a hollow on seed coat from former connection with the ovary wall of the funiculus. Plasma treatment produces the increase of the intensity of the pattern structure of the upper, epidermal layer of seed coat. Even at those magnifications, the seed surface seems to be more creased what can be interpreted in terms of the increase of micro-roughness. The effect depends on the exposure time. Exposure time of *Lavatera* seed to plasma for 2 min. results in more distinct features of the epidermal structure pattern of seed cover observed.

**Fig 5:**
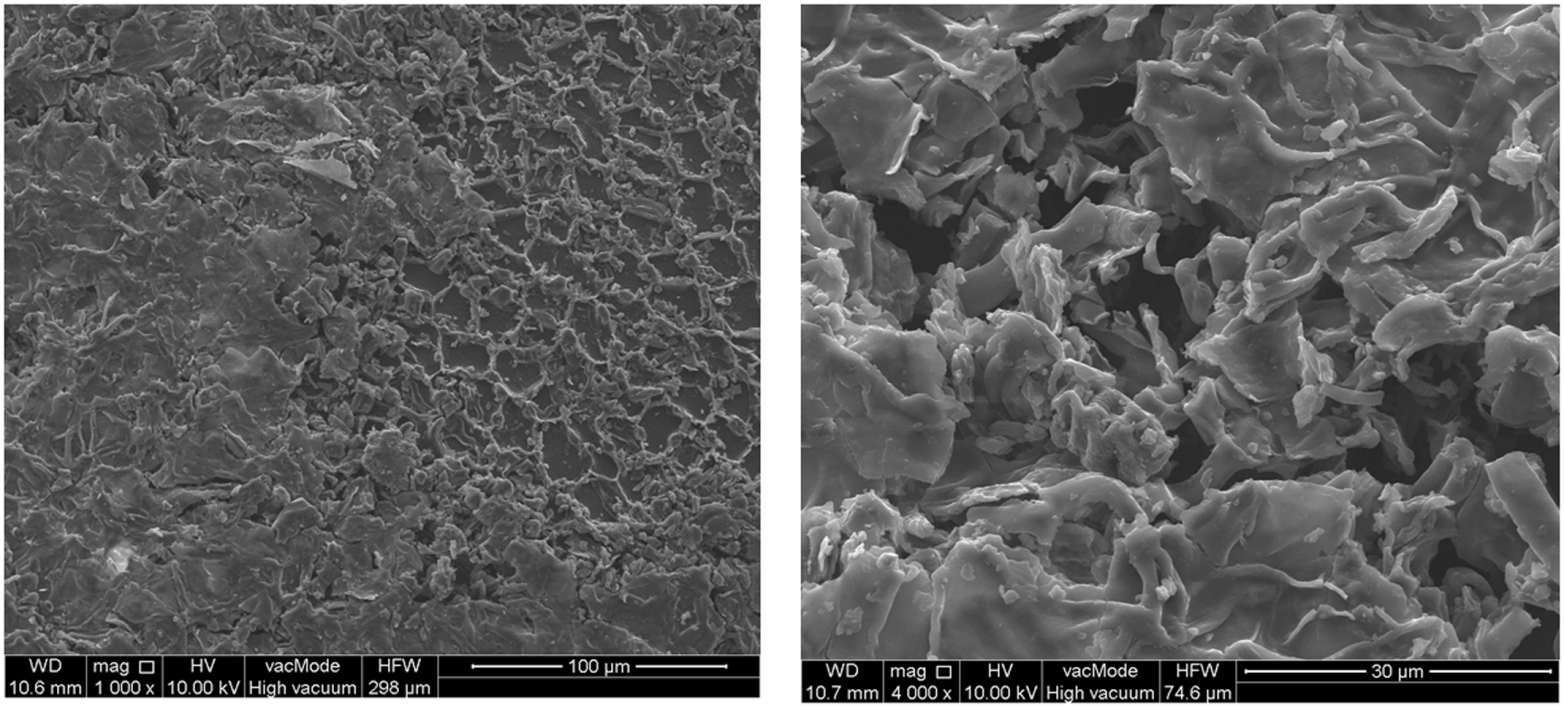
Selected *Lavatera thuringiaca* L. seed treated for 10 min with DBD plasma jet, SEM photo.

Longer exposure of seeds to plasma produce different effects. Firstly, the sharpening of the structure is observed, followed by more serious changes including damage of the parts of seed coat at peripheral sides of the seeds (at plasma exposure times as long as 10 min.)—even breakage of seed and serious changes in morphology of seed coat (Fig 5). The effect of sharpening of the seed coat structure upon plasma treatment is more visible at higher magnification, where possibly the pattern is visible more clearly. Interestingly the effect of treatment with the DBD plasma jet seems to be more pronounced that in the case while GlidArc plasma was applied. This was also confirmed by the higher germination parameters obtained in this study as compared to the reports on germination of this plant after treatment with GlidArc plasma [63].

Upon observation of more distinguished structure pattern one can suppose that DBD plasma jet treatment might destruct not only the outer surface of cuticle layer but also its inner zone. Change in the structure of the seed surface may enhance formation of micro-pores which may be involved in holding water. This would enhance the process of seed germination observed in our case. Similarly, easier water penetration to the inner seed layers may be facilitated, resulting in better seed germination. Thus pre-sowing plasma treatment may be equivalent to mechanical scarification of seeds.

## Conclusions

The study on the effect of pre-sowing DBD plasma jet treatment of *Lavatera thuringiaca* L. seeds showed the increase of germination parameters of the stimulated seeds.

Germination capacity of control seeds was 36.25% and it increased to 61.5% after 2 min. plasma treatment and to 65% after a plasma stimulation time of 5 min.

Germination energy for control amounted to 30% and became 55.5% and 56% after 2 min. and 5 min. of plasma treatment, respectively.

Analysis of the data showed a statistically significant impact of DBD plasma jet treatment on the seeds germination parameters of Thuringian Mallow in the case of exposure times of 2 and 5 min. as compared to control.

No distinguished changes in the water contact angle on the surface of the seeds were observed.

Analysis of the SEM scans revealed the increase in seed pattern intensity which could be attributed to removing of the upper cuticle layers possibly covered with wax upon short time- 2 min of plasma treatment. Longer exposure of seeds to plasma resulted in affecting the more inner parts of cuticle and even damage or fracture of some parts of the cuticle.

## Supporting information

S1 File DBD plasma jet treatment of seeds. (XLS)
